# Reduced NOV/CCN3 Expression Limits Inflammation and Interstitial Renal Fibrosis after Obstructive Nephropathy in Mice

**DOI:** 10.1371/journal.pone.0137876

**Published:** 2015-09-14

**Authors:** Pierre-Olivier Marchal, Panagiotis Kavvadas, Ahmed Abed, Chantal Kazazian, Florence Authier, Haruhiko Koseki, Shuichi Hiraoka, Jean-Jacques Boffa, Cécile Martinerie, Christos E. Chadjichristos

**Affiliations:** 1 INSERM, UMR-S938, Centre de Recherche Saint-Antoine, Saint-Antoine Hospital, Paris, France; 2 INSERM UMR-S1155, Tenon Hospital, Paris, France; 3 Sorbonne Universités, UPMC Univ Paris 6, Paris, France; 4 RIKEN Research Center for Allergy and Immunology (RCAI), RIKEN Yokohama Institute, Yokohama, Japan; 5 Department of Biochemistry, Kobe Pharmaceutical University, Kobe, Japan; 6 Department of Nephrology, Tenon Hospital, Paris, France; University of Utah School of Medicine, UNITED STATES

## Abstract

The main hallmark of chronic kidney disease (CKD) is excessive inflammation leading to interstitial tissue fibrosis. It has been recently reported that NOV/CCN3 could be involved in kidney damage but its role in the progression of nephropathies is poorly known. NOV/CCN3 is a secreted multifunctional protein belonging to the CCN family involved in different physiological and pathological processes such as angiogenesis, inflammation and cancers. The purpose of our study was to determine the role of NOV/CCN3 in renal inflammation and fibrosis related to primitive tubulointerstitial injury. After unilateral ureteral obstruction (UUO), renal histology and real-time PCR were performed in NOV/CCN3-/- and wild type mice. NOV/CCN3 mRNA expression was increased in the obstructed kidneys in the early stages of the obstructive nephropathy. Interestingly, plasmatic levels of NOV/CCN3 were strongly induced after 7 days of UUO and the injection of recombinant NOV/CCN3 protein in healthy mice significantly increased CCL2 mRNA levels. Furthermore, after 7 days of UUO NOV/CCN3-/- mice displayed reduced proinflammatory cytokines and adhesion markers expression leading to restricted accumulation of interstitial monocytes, in comparison with their wild type littermates. Consequently, in NOV/CCN3-/- mice interstitial renal fibrosis was blunted after 15 days of UUO. In agreement with our experimental data, NOV/CCN3 expression was highly increased in biopsies of patients with tubulointerstitial nephritis. Thus, the inhibition of NOV/CCN3 may represent a novel target for the progression of renal diseases.

## Introduction

The number of patients affected by CKD is growing continuously worldwide. Regardless of the primary cause of CKD, chronic inflammation and renal fibrosis are the final common pathways leading to renal structural damage [1]. The inflammatory state is initiated by the synthesis of different cytokines and chemokines promoting the upregulation of cell adhesion molecules which are required for inflammatory cell adhesion and transmigration into the damaged tissue [2–5]. The consequence of the above mentioned phenomenon is an excessive accumulation and deposit of extracellular matrix (ECM) components, leading to complete destruction of the kidney and renal failure. Among the many factors involved in renal fibrosis, angiotensin II (AngII) and transforming growth factor-beta (TGF-beta) play a crucial role **[** 6, 7]. Despite combined therapies targeting mainly the renin-angiotensin-aldosterone system renal function still declines, emphasizing the need of new therapeutic strategies.

So far, recent studies showed that the Nephroblastoma OVerexpressed gene (NOV/CCN3) could be an interesting candidate. NOV is a founder of the CCN family, a multitasking matricial proteins which are key players in several physiopathological processes such as organogenesis, inflammation, tissue repair and cancer [8, 9]. *In vitro* studies showed that NOV can regulate cell adhesion, migration, proliferation, differentiation [8–10] and survival [11, 12]. It may also affect the expression of some inflammatory mediators through direct binding to specific integrins.

During nephrogenesis in humans, NOV was found to be expressed in several renal segments. [13, 14]. In mature kidneys, its expression is low and detectable mainly in podocytes and collecting ducts [13–15]. In contrast, NOV is aberrantly expressed in nephroblastomas [13, 16, 17] and in renal cell carcinoma [18]. Recently, NOV was reported to attenuate the up-regulation of Connective tissue growth factor (CTGF/CCN2) expression induced by TGF-β1 in mesangial cells [19]. Finally, NOV was shown to have pro-angiogenic and anti-mesangioproliferative effects in anti-Thy1.1 model of glomerulonephritis in rats [20]. The above mentioned studies allowed us to speculate that NOV may play a role in the progression of CKD. For this purpose our objective was to analyze the role of NOV in a tubulointerstitial model of inflammatory renal disease induced by unilateral ureteral obstruction in mice. Our results demonstrate for the first time that the reduction of NOV expression limited inflammatory cell infiltration as well as renal interstitial fibrosis during the progression of obstructive nephropathy, indicating that this protein may represent a new therapeutic target for the progression of CKD.

## Methods

All animal work was approved by the appropriate committee of INSERM (Institut national de santé et de la recherché medicale) and the University Pierre et Marie Curie (B-75-12-01) and by the Committee on the Ethics of Animal experiments, Paris (N° Ce5/2012/091).

### Animals

Experiments were performed in 3 month old NOV-/- and wild-type littermates (WT) male mice (C57BL/6J hybrids). UUO surgery was performed under general anesthesia after intraperitoneal injection of ketamine (75–95 mg/kg) and xylazine (4–8 mg/kg). The left ureter was ligated at two separated points through a left-flank incision. Mice were sacrificed after deep anesthesia with pentobarbital after 3, 7 and 15 days and tissues were collected. Kidneys were removed, decapsulated and cut in two halves. The first half was used to performed immunohistochemistry and from the second half the cortex was dissected to perform RNA and protein extraction. Mouse peripheral blood was collected to assess NOV expression by an ELISA kit (R&D Systems).

In separate experiments, recombinant human NOV protein was produced as previously described [12, 21], and injected (50μg/mice) in 3 month old males. Mice were sacrificed 24h later.

All animals were handled in strict accordance with good animal practice as defined by the relevant national animal welfare bodies of France, and all animal work was approved by the appropriate committees as already stated at the beginning of the methods section.

### Immunohistochemistry

Kidneys were fixed in formalin solution (4%) and embedded in paraffin after conventional processing. Immunostainings for F4/80 (1:1000, Serotec) and FSP-1 [22] were performed on paraffin sections (4μm) as previously described [23].

Immunostaining for NOV (1:50) was performed on human biopsies on paraffin sections (4μm) using the Biogenex IHC kit. We used the home-made affinity-purified rabbit anti-human NOV antibodies (referred as K19M). For NOV immunofluorescence we used a home-made affinity-purified rabbit (referred as CT-Mu, anti-mouse NOV antibodies) [24].

### Western blotting

Western blotting of proteins extracted from renal cortex was performed using a commercial antibody against VCAM-1 (1:1000, Abcam). GAPDH (1:10000, Sigma-Aldrich) was used as loading controls.

### RNA extraction and RT-PCR

Total RNA was extracted from renal cortex using the Nucleospin RNA II kit (Macherey-Nagel). Reverse transcription and semi-quantitative real-time PCR amplification on the ABI 7300 apparatus (Applied Biosystems) were performed as previously described [25]. Specific primers (Proligo, Sigma-Aldrich) used for amplification of target genes were designed using the Primer3 Input program and are presented in Table 1. The comparative Ct method [26] was used to calculate gene expression values. The ribosomal S26 was used as housekeeping gene. PCR amplifications were assessed from two reverse transcriptions per experiment.

**Table 1:** Primers sequences.

Genes	Sense primers	Antisense primers
**NOV/CCN3**	CTGCATTGAACAGACCACAGA	TCTTGAACTGCAGGTGGATG
**MCP-1/CCL2**	AGGTCCCTGTCATGCTTCTG	TCTGGACCCATTCCTTCTTG
**VCAM-1**	CCCGTCATTGAGGATATTGG	GGTCATTGTCACAGCACCAC
**IL-6**	CCAATTTCCAATGCTCTCCT	ACCACAGTGAGGAATGTCCA
**F4/80**	TGAATGGCTCCATTTGTGAA	GGCCCTCCTCCACTAGATTC
**CD68**	TCCAAGCCCAAATTCAAATC	ATTGTATTCCACCGCCATGT
**Col1**	CCCGAGGTATGCTTGATCTG	GGGTCCCTCGACTCCTACAT
**CTGF**	TGACCTGGAGGAAAACATTAAGA	AGCCCTGTATGTCTTCACACTG
**S26**	AAGGAGAAACAACGGTCGTG	AGAGCTTGGGAAGCACGTAA

### Statistical analysis

Data were expressed as mean ± SEM. Results were analyzed using one-way analysis of variance followed by protected least significant difference Fisher’s test of the Stat-view software package. Results with P<0.05 were considered statistically significant.

## Results

### NOV expression is increased in the obstructed kidneys and plasma during the progression of obstructive nephropathy in mice

**Fig 1:**
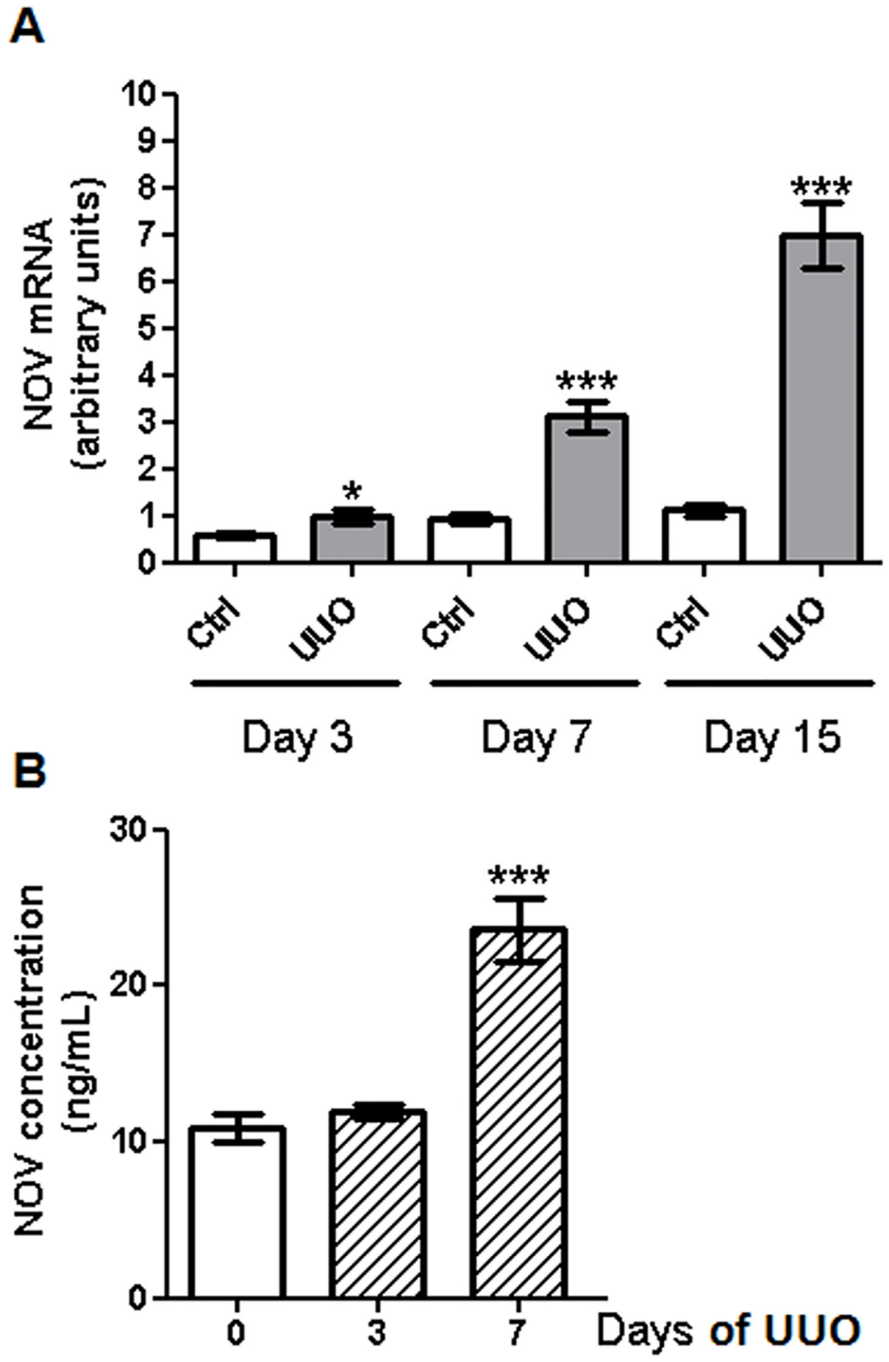
NOV expression in renal cortex and plasma after UUO. (A) RT-qPCR analysis in the renal cortex showed increased expression of NOV mRNA after 3, 7 and 15 days of UUO compared to contralateral cortex. Values are means ± SEM and expressed as the ratio of the target gene to the internal control gene (S26 rRNA). n = 7 for each group. *P<0.05 and ***P<0.001, UUO vs. ctrl, #P<0.05, WT ctrl versus Nov-/- ctrl. (B) Quantification of NOV concentration by ELISA showed an increased expression of NOV in plasma after 7 days of UUO compared to sham mice. Values are means ± SEM in ng/mL. n = 9 for sham animals and 4 for both UUO time points. ***P <0.001, UUO vs. sham.

To determine whether NOV could be involved in obstructive nephropathy, we checked its expression after 3, 7 and 15 days of UUO. mRNA levels of NOV were significantly increased over time (by ~1.7 fold at 3 days, ~3.4 fold at 7 days and ~6.3 fold at 15 days) in the renal cortex of obstructed kidneys compared to contralateral ones (Fig 1A). No significant differences were observed between contralateral kidneys at different time points. As NOV is a systemic circulating protein [27] we measured its concentration in plasma after UUO. We observed a significant increase of the plasmatic levels of NOV (by~2.2 fold) after 7 days of UUO compared to control mice (Fig 1B). At basal conditions NOV was mainly detected in the glomeruli and the vascular media. After 7 days of UUO *de novo* expression of NOV was observed in the tubulointerstitial compartment and mainly in tubular cells (Figure A in S1 File). These results demonstrate that NOV expression is increased in damaged kidneys as well as in plasma of mice after the induction of obstructive nephropathy.

### NOV modulates the expression of inflammatory markers after UUO

**Fig 2:**
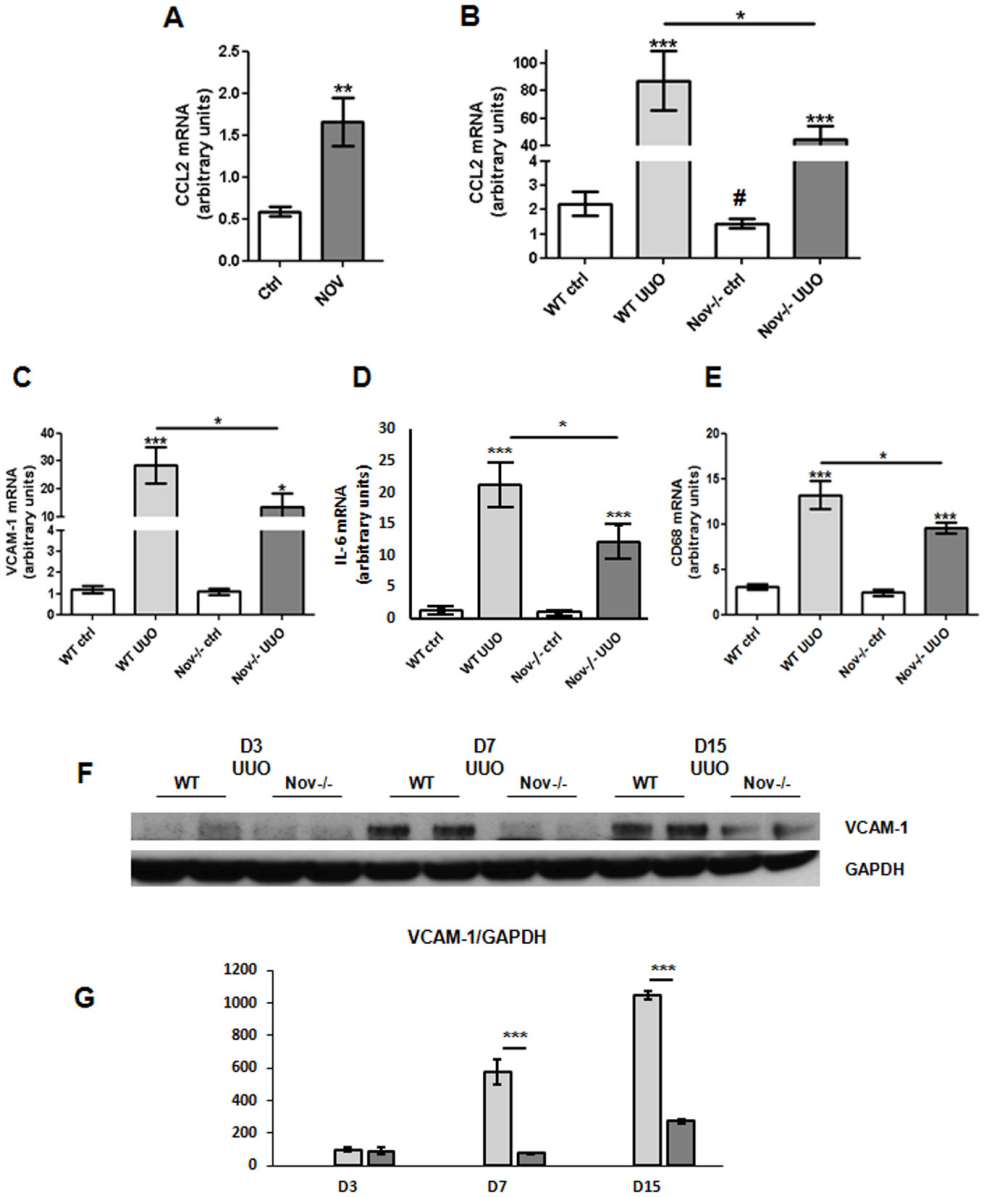
Inflammatory markers expression after injection of NOV recombinant protein and/or in absence of NOV after 3 days of UUO. (A) qPCR analysis in the renal cortex showed increased expression of CCL2 mRNA 24h after injection of NOV recombinant protein (50μg/mouse) compared to mice injected with vehicle. n = 4 for each group. **P<0.01, NOV treated mice vs. vehicle. (B), (C), (D) and (E) qPCR analysis in the renal cortex showed a marked increase of CCL2 (B), VCAM-1 (C), IL6 (D) and CD68 (E) mRNAs expression after 3 days of UUO in WT. In Nov-/-, upregulation of the above mentioned molecules was significantly restricted. Values are means ± SEM and expressed as the ratio of the target gene to the internal control gene (S26 rRNA). n = 9 for WT and 6 for NOV-/-. *P<0.05, **P<0.01 and ***P<0.001, UUO vs. ctrl. (F) (G) Representative western blots for VCAM-1 and GAPDH were performed by using renal cortex of contralateral controls and obstructed kidneys from WT and NOV-/- mice. Graphs show quantification of western blots expressed as the ratio of VCAM-1 versus GAPDH signal for each sample (G).

**Fig 3:**
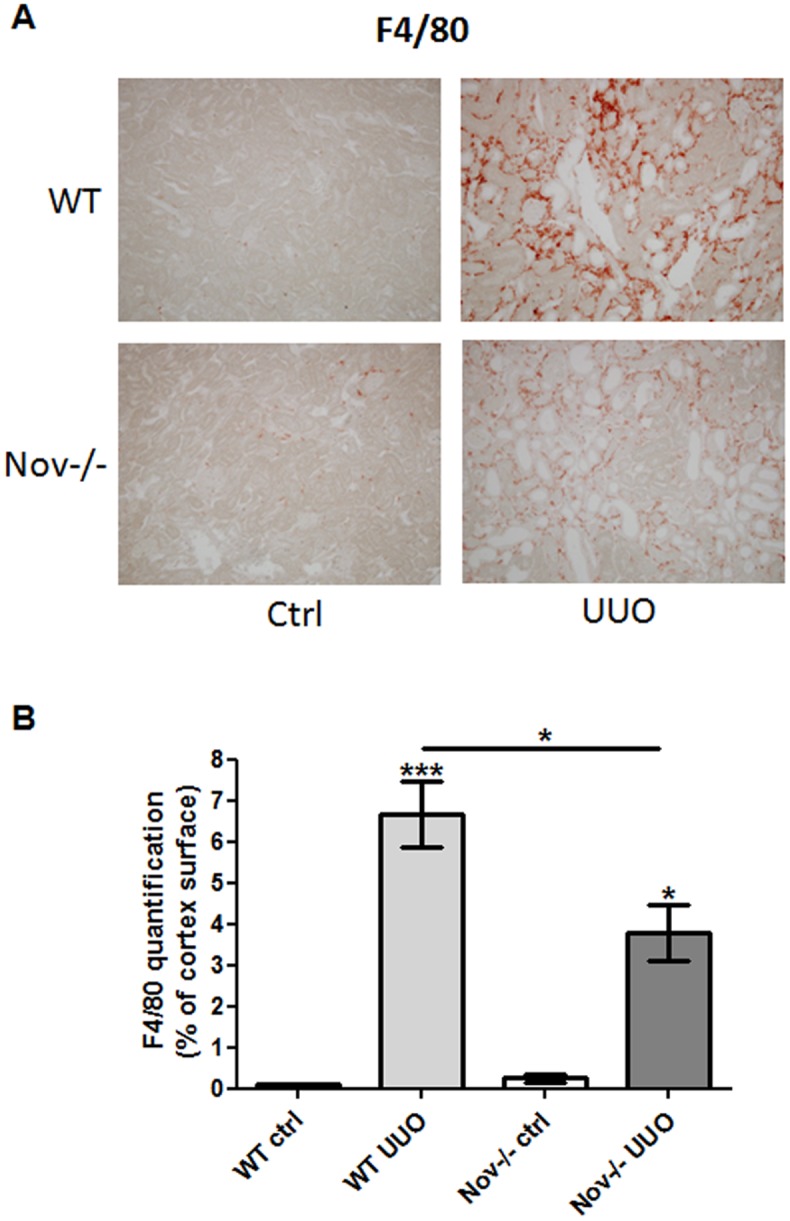
F4/80 expression in absence of NOV after 7 days of UUO. (A) Immunostaining and (B) quantification showed a marked increase of F4/80 protein expression after 7 days of UUO in WT. In Nov-/-, the upregulation of F4/80 was significantly restricted. Values are means ± SEM expressed as arbitrary units. n = 3 for ctrl, 9 for WT UUO and 7 for NOV-/- UUO. *P<0.05, UUO vs. ctrl. Magnification X200 (A).

We have previously reported *in vivo* that NOV induced CCL2 expression, a key player in inflammatory cells recruitment, after an unilateral injection of recombinant NOV protein into the cerebral cortex [28]. Consequently, we hypothesized that NOV could also regulate CCL2 expression in the kidney. We first performed an intraperitoneal injection of NOV recombinant protein in healthy mice and sacrificed them after 24 hours. As evidenced by real-time PCR CCL2 expression was increased by almost 3-fold within kidneys (Fig 2A). Next, we analyzed the impact of the absence of NOV on some inflammatory markers expression on NOV-/- and littermates during the progression of obstructive nephropathy. Expression of CCL2 (Fig 2B), VCAM-1 (Fig 2C), IL-6 (Fig 2D) and CD68 (Fig 2E) mRNA were highly increased in the obstructed kidneys of WT mice after 3 days of UUO. In contrast, at the same time point their levels were significantly reduced in NOV-/- mice. No significant differences were detected between NOV-/- and WT mice in the contralateral kidneys except for CCL2 mRNA as its expression was significantly higher in WT controls compared with Nov-/- controls. Furthermore, western blots confirmed that VCAM-1 upregulation was blunted in real cortexes of NOV-/- mice after 7 and 15 days of UUO (Fig 2F and 2G). Since monocytes play a major role in the inflammatory process in this model of renal nephropathy [29], we next performed F4-80 immunostainings. No staining was observed in contralateral renal cortical slides of WT and NOV-/- mice. A strong infiltration of F4/80 positive immune cells was detected within the interstitium of the obstructed kidneys of the WT mice 7 days after UUO, whereas in NOV-/- mice the infiltrate was significantly reduced (Fig 3A and 3B). Our data strongly suggest that decreased expression of NOV limited the inflammatory reaction during the progression of obstructive nephropathy.

### Decreased NOV expression reduces interstitial fibrosis following obstructive nephropathy

**Fig 4:**
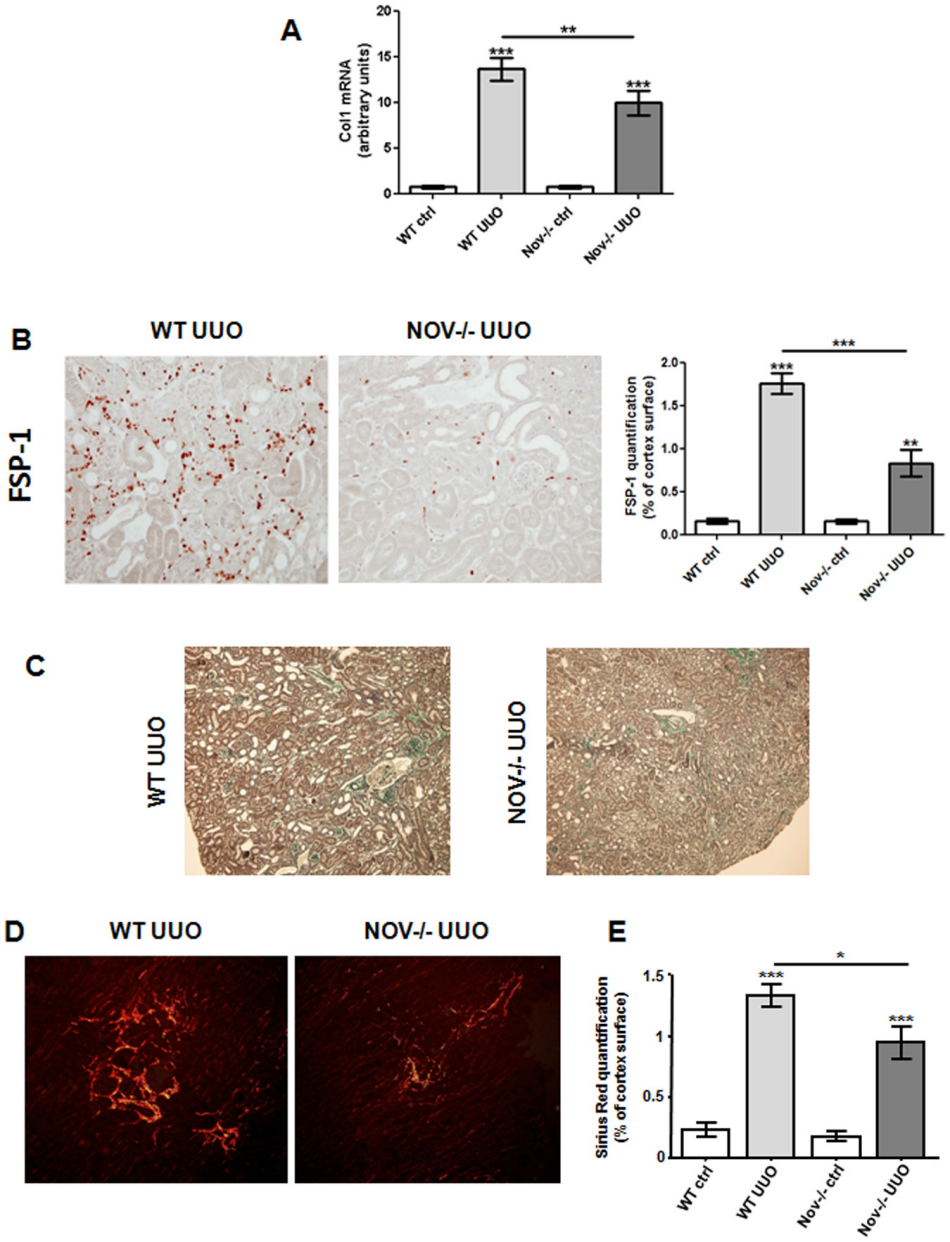
Expression of fibrotic markers in absence of NOV after UUO. (A) qPCR analysis in the renal cortex showed a marked increase of Col1 mRNA expression after 7 days of UUO in WT. In NOV-/-, the upregulation of Col1 was significantly restricted. Values are means ± SEM and expressed as the ratio of the target gene to the internal control gene (S26 rRNA). n = 9 for WT and 6 for NOV-/-. **P<0.01 and ***P<0.001, UUO vs. ctrl. (B) Immunostaining and quantification showed a marked increase of FSP-1 protein expression after 7 days of UUO in WT. In NOV-/-, the upregulation of FSP-1 was blunted. Values are means ± SEM expressed as arbitrary units. n = 3 for ctrl and 6 for UUO. **P<0.01 and ***P<0.001, UUO vs. ctrl. Magnification X200. (C) Representative examples of renal cortical histology revealed by Masson’s trichrome in WT and NOV-/- mice after 7 days of UUO. Magnification X200. (D) Sirius Red staining and (E) quantification showed a marked increase of interstitial collagen expression after 15 days of UUO in WT. In NOV-/-, renal fibrosis was significantly restricted. Values are means ± SEM expressed as arbitrary units. n = 5 for ctrl and 6 for UUO. *P<0.05, **P<0.01 and ***P<0.001, UUO vs. ctrl.

Given that NOV-/- were protected against inflammation, we studied the impact of NOV deletion on the progression of renal interstitial fibrosis after UUO. We observed that Col1 mRNA was significantly increased in ligated kidneys compared to contralateral ones after 7 days of UUO (Fig 4A). However, this upregulation was blunted in NOV-/-. In addition, at the same time point, immunostaining for Fibroblast specific protein-1 (FSP-1), a widely established fibrotic marker, showed limited FSP-1-positive cells after UUO in NOV-/- mice (Fig 4B). Furthermore, as illustrated in Fig 4C, Masson’s Trichrome showed a substantial improvement of the renal structure of the NOV-/- mice compared to the WT ones. Finally, in accordance with our previous observations, accumulation of interstitial collagen, assessed by Sirius Red coloration, was significantly reduced after 15 days of UUO (Fig 4D and 4E).

In conclusion, our data strongly suggest that decreased expression of NOV limited the inflammatory reaction and decreased the excessive extracellular matrix accumulation leading to improved renal structure in the UUO model.

### NOV is de novo expressed within renal interstitium in patients with tubulointerstitial nephropathy

**Fig 5:**
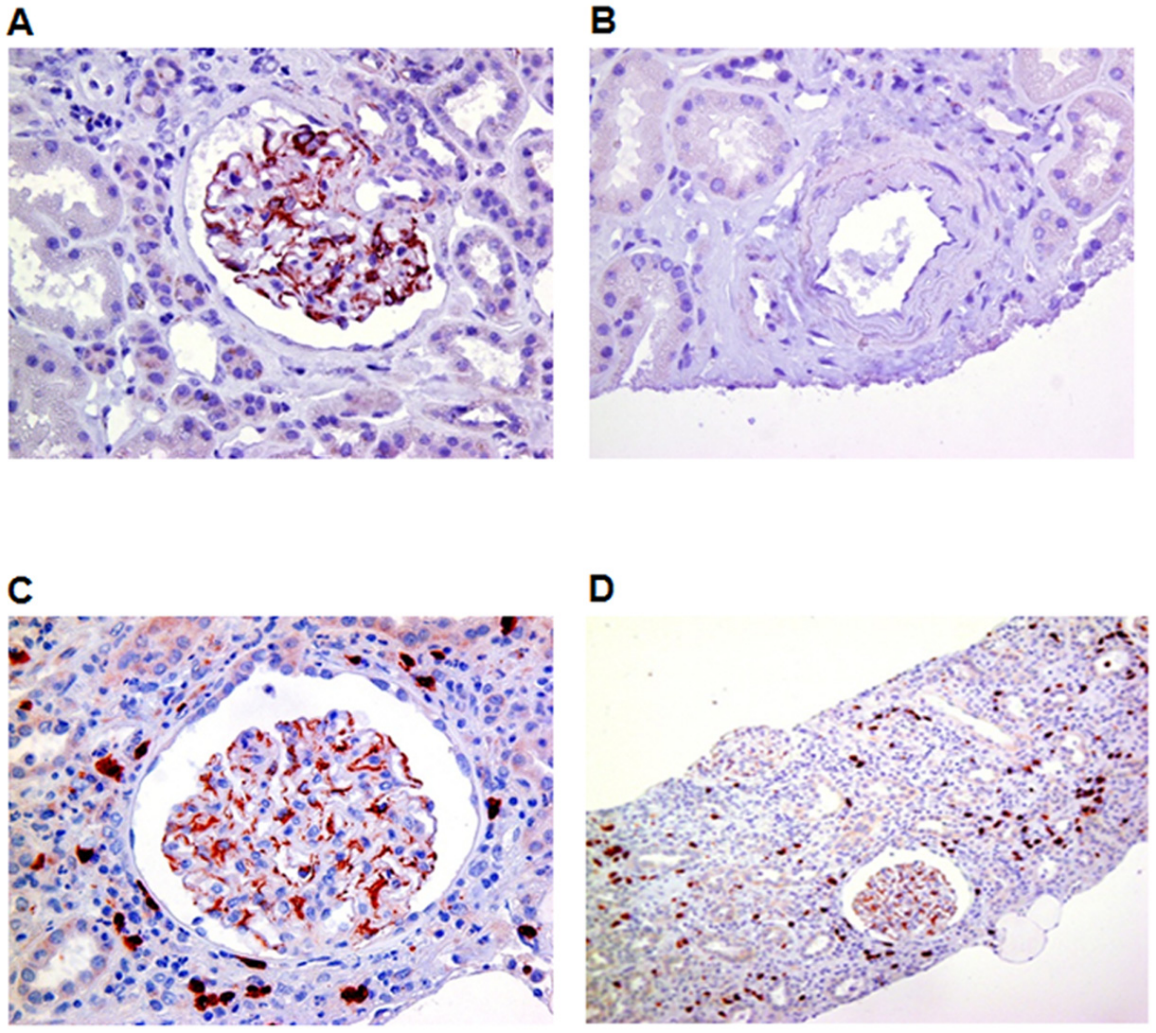
NOV expression in human tubulointerstitial nephritis. (A), (B), (C) and (D) Representative immunostainings of human biopsies showed a glomerular expression of NOV in healthy kidneys (A and B). Tubulointerstitial nephritis induced a de novo expression of NOV within the tubulointerstitial compartment (C and D). Magnification X400 (A, B and C) and X200 (D).

Since NOV expression was upregulated in mice during the progression of obstructive nephropathy, we studied its expression in patients suffering from tubulointerstitial nephropathy. In control biopsies NOV was mainly expressed within glomeruli probably in podocytes as already described [15] (Fig 5A), whereas it was almost absent in the interstitium and renal vessels (Fig 5B). In biopsies from tubulointerstitial nephritis patients we observed a de novo expression of NOV within the interstitial compartment (Fig 5C and 5D). These data clearly demonstrate that NOV is *de novo* expressed in human tubulointerstitial injury.

## Discussion

In this study, we used the UUO model of experimental nephropathy which is well known to initiate a rapid sequence of events in the obstructed kidney, leading to huge inflammatory response, tubular cell death and irreversible hydronephrosis [30].

We showed that, among other inflammatory markers, NOV expression increased during the progression of obstructive nephropathy and NOV-/- mice displayed limited inflammation and renal interstitial fibrosis during the progression of the disease. To our knowledge this is the first time that NOV is described as a deleterious molecule in the experimental nephropathy by using NOV-/- mice. In addition, in basal conditions these mice, seemed to have a normal phenotype as renal structure and function were identical compared to wild type littermates.

The first observation we made was that NOV expression was increased within the renal cortex at the early stages of the obstructive nephropathy. Its mRNA expression pattern was paralleled closely by that of the inflammatory and cell adhesion markers suggesting that NOV may participate in the recruitment of inflammatory cells within the injured kidneys. Interestingly, the administration of NOV recombinant protein in WT mice increased the expression of MCP1/CCL2, a major chemoattractant for monocytes. In addition, CCL2 mRNA expression was significantly higher in control kidneys of WT mice compared with Nov-/- ones, reinforcing our hypothesis that NOV could influence the expression of this inflammatory marker. However this difference was attenuated in the contralateral kidneys at latter time points while in the UUO groups the decrease of CCL2 mRNA in the Nov-/- was of borderline significance (Figure B in S1 File). However, it should be taken into consideration that this is the mRNA level while at the same time point inflammatory infiltrates were highly decreased in the Nov-/- kidneys (Fig 3). Thus, the overexpression of NOV during the progression of the disease after UUO may amplify the inflammatory process. The implication of NOV in this phenomenon is poorly known. Consistent with our study, systemic overexpression of NOV increased the expression of MCP-1/CCL2 and regulated on activation normal T cell expressed and secreted (RANTES), within glomeruli in rats with progressive anti-Thy1.1 nephritis [20]. In addition, we have previously reported that intracranial injection of recombinant NOV in mice significantly increased the expression of both CCL2 and CXCL1 in astrocytes [28]. These proinflammatory effects seemed to be mediated via the integrins β1 and β5 respectively. Similar results were recently obtained by our team by incubation adipocytes with NOV recombinant protein (unpublished observations). In contrast, other studies showed that adenoviral overexpression of CCN3 in HUVECs markedly reduced the cytokine-mediated induction of VCAM-1 and further monocyte adhesion, by inhibiting the nuclear accumulation of the nuclear factor kappaB (NF-κB) [31]. Therefore, based on the above mentioned studies NOV may have both pro- and anti-inflammatory properties. These differences could reflect various cellular adaptive processes that take place in distinct experimental models. Indeed, some studies demonstrated that NOV itself can be modulated by proinflammatory cytokines, and can then regulate the expression of chemokines via distinct signaling pathways. Additional studies using NOV-/- mice in other models of experimental nephropathy will be of particular interest to determine whether the anti-inflammatory effect of the NOV blockade is model dependent or it can be considered as a more common protective mechanism of renal disease.

Extracellular matrix remodeling is a highly dynamic process occurring in response to inflammation. Our study clearly demonstrated that NOV had profibrotic properties as renal interstitial fibrosis was inhibited in NOV-/- mice during the progression of obstructive nephropathy. The implication of NOV in the fibrotic process is a matter of debate. It has been reported that in contrast to other members of the CCN family, NOV acted against the development of fibrosis. Indeed, an inverse correlation between NOV and CTGF has been described in several *in vitro* and *in vivo* experimental models, suggesting that these two CCN members could have antagonistic functions [9]. In addition, we have recently reported in a hypertensive model of experimental nephropathy in mice, that increased expression of NOV could prevent the up regulation of CTGF within damaged kidneys, at the early stages of the disease, whereas at the latter stages NOV subsequent down-regulation could contribute to CTGF induction. This hypothesis was reinforced by the fact that overexpression of NOV in vascular smooth muscle cells inhibited the angiotensin II profibrotic effects by down regulating both AT_1_R and CTGF expressions (Marchal PO et al., 2014 submitted manuscript). These observations are in line with previous data showing that the suppression of NOV enhanced expression of profibrotic marker proteins, such as α-smooth muscle actin, collagen type I, and CTGF in primary rat hepatic stellate cells [32]. In addition, overexpression of NOV reduced glomerulosclerosis and cortical accumulation of collagen type I in progressive glomerulonephritis [20]. Finally, recent studies have also shown that NOV down-regulated CTGF in mesangial cells [19] underlining a direct relation between these molecules. However, in our model of obstructive nephropathy we haven’t noticed any significant difference between NOV-/- and WT mice even if CTGF expression was increased in both genotypes during the progression of obstructive nephropathy (Figure C in S1 File). These differences could be explained by the fact that the biological functions of NOV may be highly dependent on the environment in which this protein is expressed. In addition, the nature of the interactions in distinct physiopathological contexts can also govern the ability of NOV to interact with various partners providing contradictory results. Of note, recent studies showed that adenoviral NOV gene transfer failed to attenuate fibrogenesis in an *in vivo* model of experimental liver fibrosis [32].

Another major finding of our study is that plasmatic levels of NOV were highly upregulated in WT mice after 7 days of UUO. This is of major interest because all biological parameters related to renal function were normal in this model of experimental nephropathy, as the contralateral kidney preserved a normal renal function. This observation suggests that NOV could be used as a biomarker to predict renal damage at the early stages of chronic kidney disease. It will be of interest to check this possibility by correlating plasmatic and urinary levels of NOV in additional models of experimental nephropathy, in which renal function is decreased during the progression of the disease. Interestingly, we observed an upregulation of NOV expression in biopsies of patients suffering from tubulointestitial diseases, a pathological situation that is close to our experimental study. Consequently, it would also be of interest to investigate whether the expression of NOV is correlated with the progression of human renal disease. This will determine whether tissue and/or plasma NOV can be used as new molecular markers to identify patients prone to develop renal disease. Interestingly, a recent study reported that plasma levels of NOV were closely associated with obesity in patients suffering from metabolic disorders [33].

In summary, our data demonstrated that NOV overexpression in response to injury may participate in inflammation and tissue fibrosis in CKD. Although further work is required to decipher the molecular mechanisms involving NOV in renal inflammatory diseases, our study indicates for the first time that this protein may represent a new therapeutic target against the progression of CKD.

## Supporting Information

S1 File Figure A in S1 File. Progressive increase in NOV expression in mice after UUO, Figure B in S1 File. mRNA expression of CCL2 in mice after 7 days of UUO, and Figure C in S1 File. mRNA expression of CTGF in mice after UUO.
(PDF)
