# Supplementary material for: Reduced NOV/CCN3 Expression Limits Inflammation and Interstitial Renal Fibrosis after Obstructive Nephropathy in Mice
Source: PLoS One. 2015 Sep 14;10(9):e0137876. doi: 10.1371/journal.pone.0137876 (PMC4569074; doi:10.1371/journal.pone.0137876)
Supplement: S1 File — Figure A in S1 File. Progressive increase in NOV expression in mice after UUO, Figure B in S1 File. mRNA expression of CCL2 in mice after 7 days of UUO, and Figure C in S1 File. mRNA expression of CTGF in mice after UUO. (PDF) [file pone.0137876.s001.pdf]

Figure A. **Progressive increase in NOV expression in mice after UUO.**

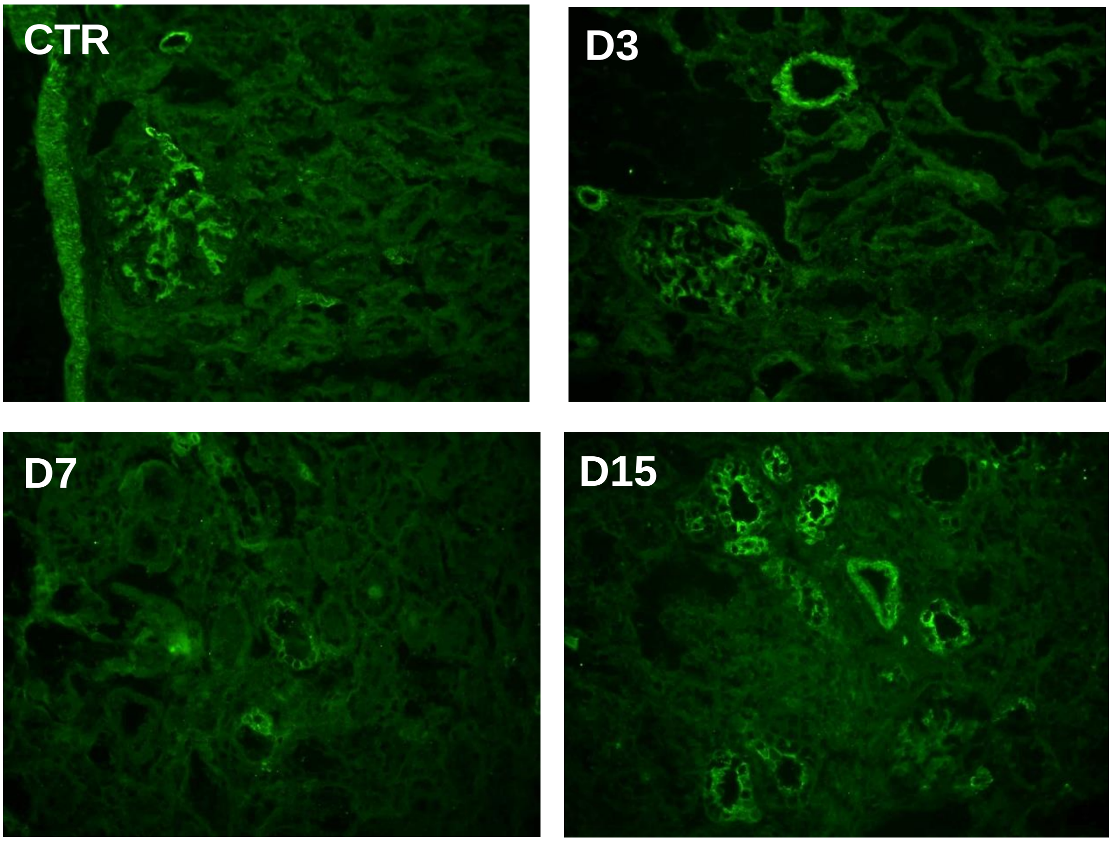

Figure B. mRNA expression of CCL2 in mice after 7 days of UUO.

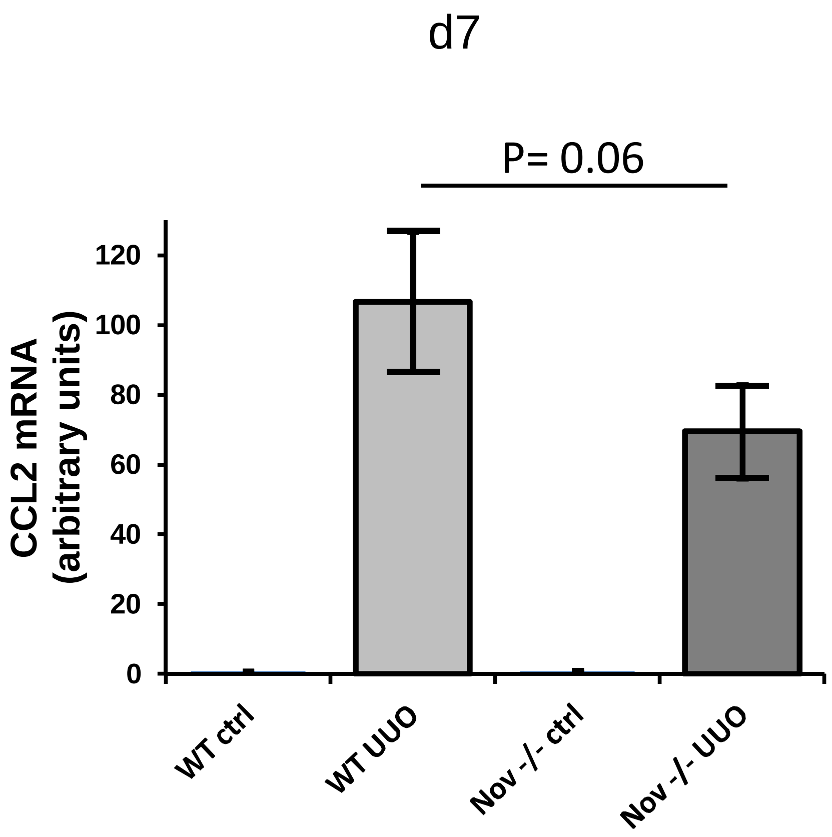

Figure C. **mRNA expression of CTGF in mice after UUO.**

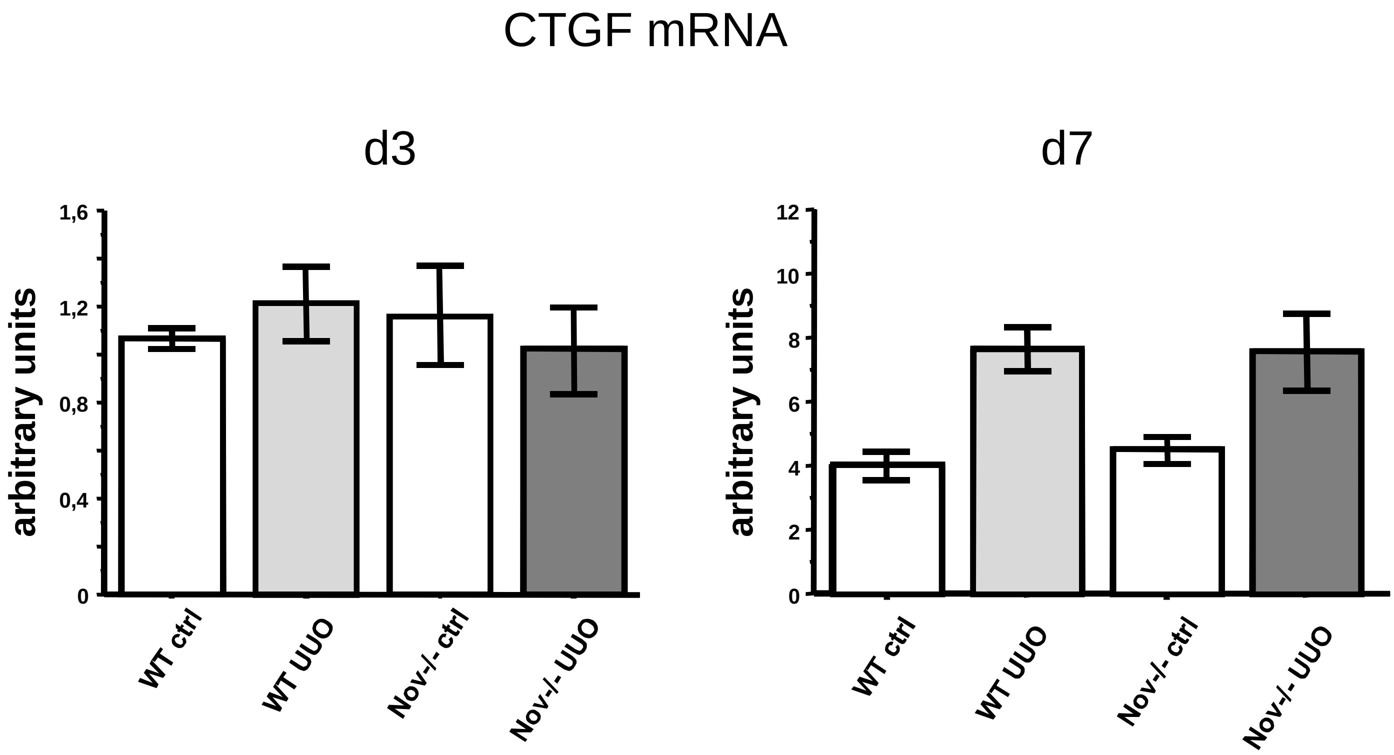

Supplementary figure legends.

Fig A. Progressive increase in NOV expression in mice after UUO.

NOV immunostainings (green) were performed on cryosections from renal cortical slides of controls and mice after 3, 6 and 15 days of UUO. In basal conditions NOV was expression was detected in the glomerular and vascular compartments. NOV was de novo expressed in the renal tubules from 6 days of obstructive nephropathy. Photographs are representative of kidneys from 6 mice at each time point in both groups. Magnification of microphotographs: X400.

Fig B. mRNA expression of CCL2 in mice after 7 days of UUO.

qPCR showed that CCL2 mRNA expression in NOV-/- and WT mice in basal conditions and 7 days of UUO. Values are expressed as mean ± SEM, n=6 (triplicates).

Fig C. mRNA expression of CTGF in mice after UUO.

qPCR showed that CTGF mRNA expression was not found to be different between NOV-/- and WT mice in basal conditions or after 3 and 7 days of UUO. Values are expressed as mean ± SEM, n=6 (triplicates), * P<0.05, **P<0.01, ***P<0.001).
